# Intravitreal Faricimab Prior to Direct Photocoagulation Improves Anatomical Outcomes in Focal Diabetic Macular Edema

**DOI:** 10.3390/jcm15093487

**Published:** 2026-05-02

**Authors:** Yuki Sanada, Yoshihiro Takamura, Yutaka Yamada, Hideyuki Oshima, Makoto Gozawa, Takehiro Matsumura, Masaru Inatani

**Affiliations:** Department of Ophthalmology, Faculty of Medical Sciences, University of Fukui, Eiheiji-cho, Yoshida-gun 910-1193, Fukui-ken, Japan; sndyk@u-fukui.ac.jp (Y.S.); twilightprincess0616@gmail.com (Y.Y.); osm@u-fukui.ac.jp (H.O.); makoto.gozawa@gmail.com (M.G.); takebou_mail@yahoo.co.jp (T.M.); inatani@u-fukui.ac.jp (M.I.)

**Keywords:** non-center involved diabetic macular edema, faricimab, direct photocoagulation, laser, anti-VEGF therapy, microaneurysm, treatment sequence

## Abstract

**Purpose:** To determine the optimal treatment sequence for combination therapy using intravitreal faricimab (IVF) and direct photocoagulation (PC) in eyes with non-center-involved diabetic macular edema (DME). **Methods:** This retrospective study included 35 eyes with focal DME treated with IVF and PC targeting microaneurysms (MAs). Treatment success was defined as resolution of focal edema, indicated by disappearance of the white area (WA) on optical coherence tomography. Eyes were assigned to a PC-IVF group (initial PC followed by IVF if edema persisted after 2 months; *n* = 20) or an IVF-PC group (initial IVF followed by PC for residual edema; *n* = 15). Additional PC was performed every 2 months as needed. **Results:** Cumulative success rates at 2, 4, and 6 months were 35.0%, 70.0%, and 90.0% in the PC-IVF group and 60.0%, 93.3%, and 100% in the IVF-PC group, respectively. Macular volume significantly decreased at all time points in the IVF-PC group (all *p* < 0.01), whereas a significant reduction was observed only after 6 months in the PC-IVF group (*p* < 0.01). The number of MAs and the extent of edema were significantly reduced after 2 months in both groups, with greater reductions in the IVF-PC group (*p* < 0.05). The number of laser shots required for initial PC was significantly lower in the IVF-PC group (*p* < 0.0001), and the mean number of PC sessions was also reduced (0.6 vs. 1.8). In the PC-IVF group, baseline edema size was significantly smaller in successfully treated eyes (*p* < 0.001). **Conclusions:** Initiating treatment with IVF prior to PC may be advantageous in focal DME, particularly in eyes with larger edema, enabling faster anatomical improvement and reducing the need for laser treatment. Direct PC alone may be sufficient for small focal lesions with limited edema, supporting an individualized treatment strategy.

## 1. Introduction

Diabetic macular edema (DME) is a leading cause of vision loss among individuals with diabetes [1]. DME is broadly classified into diffuse and focal types, both of which are associated with microaneurysms (MAs) [2]. In focal DME, MAs are typically located within areas of retinal thickening and are often accompanied by circinate hard exudates [3]. Leakage from MAs into the surrounding retinal tissue results in localized edema, and direct laser photocoagulation (PC) targeting MAs has long been established as an effective treatment modality [4,5]. In Japan, a survey of retinal specialists reported that direct PC is commonly selected as a first-line treatment for focal DME, whereas anti-vascular endothelial growth factor (VEGF) therapy is preferred for diffuse DME [6].

Advances in laser technology, such as the navigated laser system (Navilas^®^, OD-OS, Teltow, Germany), have improved the precision of PC by enabling automated eye tracking and image-guided treatment delivery [7,8]. This system achieves higher accuracy in targeting MAs than conventional manual laser techniques [9]. Furthermore, navigated PC has been shown to induce high rates of MA closure and reduce retinal thickness in patients with DME, supporting its effectiveness in focal disease [10].

In parallel, anti-VEGF therapy has become the standard of care for center-involved DME, with multiple randomized clinical trials demonstrating significant anatomical and functional improvements [11,12,13]. However, a substantial proportion of patients exhibit incomplete responses, with persistent edema despite repeated injections [14]. In such cases, residual focal edema is often associated with a high density of MAs, suggesting that these lesions contribute to treatment resistance [15,16].

Faricimab is a bispecific antibody that targets both VEGF-A and angiopoietin-2 (Ang-2), thereby providing dual inhibition of pathways involved in vascular permeability and instability [17]. Large-scale randomized clinical trials, including the YOSEMITE and RHINE studies, have demonstrated that faricimab provides significant and durable anatomical and functional improvements in patients with DME, supporting its clinical utility [18]. We previously reported that intravitreal faricimab (IVF) significantly reduces the number of MAs in eyes with DME [19]. After 3 monthly IVF injections, many MAs disappeared, and IVF not only suppressed the formation of new MAs but also reduced preexisting ones [19]. These findings indicate that IVF may not only suppress vascular leakage but also directly influence the structural components underlying focal edema.

Given that both direct PC and IVF target MAs through different mechanisms, combination therapy may offer complementary benefits in the management of focal DME. However, the optimal sequence of these treatments remains unclear. In particular, it is unknown whether initial reduction of MAs by IVF enhances the efficacy of subsequent PC, or whether early PC targeting visible MAs is sufficient before administering anti-VEGF therapy. Therefore, the purpose of this study was to determine the optimal treatment sequence for combination therapy using IVF and direct PC in eyes with non-center-involved focal DME.

## 2. Materials and Methods

### 2.1. Study Design

This retrospective comparative study adhered to the tenets of the Declaration of Helsinki and was approved by the Institutional Review Board of the University of Fukui (No. 20250136, approved on 29 September 2025). Informed consent was obtained using an opt-out approach.

### 2.2. Participants

Patients aged > 20 years with type 2 diabetes mellitus and non-center-involved focal diabetic macular edema (DME), confirmed by leakage from microaneurysms (MAs) on fluorescein angiography (FA), were enrolled between June 2022 and April 2025.

The exclusion criteria were as follows: (1) prior treatment with anti-VEGF agents, corticosteroids, or retinal photocoagulation within 6 months; (2) active intraocular inflammation or infection; (3) uncontrolled glaucoma; (4) other retinal diseases (e.g., retinal vein occlusion or retinal detachment); (5) history of stroke; (6) uncontrolled hypertension (systolic > 180 mmHg or diastolic > 100 mmHg); (7) significant media opacity affecting fundus evaluation; (8) vitreomacular traction or epiretinal membrane; and (9) proliferative diabetic retinopathy with active neovascularization.

### 2.3. Treatment Protocol

Patients were divided into two groups according to treatment sequence: the PC-IVF group and the IVF-PC group. In the PC-IVF group, direct photocoagulation (PC) was performed first. If focal edema persisted after 2 months, IVF was administered. In the IVF-PC group, IVF was performed initially. The treatment sequence was determined based on the institutional treatment protocol during different time periods. From 4 January 2023 to 31 March 2024, patients were treated with initial direct PC, followed by IVF if focal edema persisted after 2 months (PC-IVF group). From 1 April 2024 to 28 September 2025, the treatment strategy was changed to initiate therapy with IVF, followed by PC for residual edema after 2 months (IVF-PC group). In both groups, additional PC was performed at 2-month intervals in cases of persistent or recurrent edema. In the IVF-PC group, the initial IVF protocol consisted of a single intravitreal injection without a loading phase. All clinical evaluations and therapeutic procedures, including intravitreal injections and PC, were performed by a single experienced ophthalmologist (Y.T.).

### 2.4. Laser Photocoagulation Procedure

Direct PC was performed after pupil dilation and topical anesthesia using a contact lens (H-R Centralis^®^, Volk Optical, Mentor, OH, USA). A navigated laser system (Navilas^®^ 577s Prime; OD-OS, Teltow, Germany) with a wavelength of 577 nm was used.

MAs within edematous regions were identified based on merged optical coherence tomography (OCT) maps and OCT angiography images. The laser parameters were as follows: spot size, 60 μm; pulse duration, 50–100 ms; and power, 100–150 mW, adjusted to achieve a gray-white burn. These laser parameters were selected in accordance with previously reported protocols for navigated PC in DME, aiming to achieve effective MA closure while minimizing retinal damage [7].

### 2.5. Intravitreal Injection Procedure

Intravitreal injections were performed under sterile conditions using a standardized protocol. After eyelid speculum placement and disinfection with povidone iodine, faricimab (Vabysmo^®^, 2 mg/0.05 mL; Chugai Pharmaceutical Co., Ltd., Tokyo, Japan) was injected into the vitreous cavity by a trained ophthalmologist.

### 2.6. Outcome Measures

Focal edema was defined as a white area (WA) on OCT color maps measuring > 500 μm. The primary outcome was treatment success, defined as complete resolution of focal edema, indicated by disappearance of the WA on OCT color maps. Secondary outcomes included changes in macular volume, WA width, number of MAs within the WA, and number of laser shots. Best-corrected visual acuity (BCVA) and OCT parameters were evaluated at baseline and after 2, 4, and 6 months. Recurrence was defined as reappearance of WA after initial resolution.

### 2.7. Imaging and Measurements

Color fundus photographs were obtained using a fundus camera (VX-10i, Kowa, Nagoya, Japan). FA and OCT images were acquired using Spectralis OCT (Heidelberg Engineering, Heidelberg, Germany). WA width was measured using image analysis software (Adobe Photoshop CS6 Extended, 13.1.2, Adobe Systems, San Jose, CA, USA).

### 2.8. Statistical Analysis

All statistical analyses were performed using JMP software version 19 (SAS Institute Inc., Tokyo, Japan). Data are presented as mean ± standard deviation (SD). Between-group comparisons were performed using the Mann–Whitney U test, and within-group comparisons over time were analyzed using the Wilcoxon signed-rank test. A *p*-value < 0.05 was considered statistically significant.

## 3. Results

### 3.1. Patient Characteristics

A total of 35 eyes from 35 patients with focal diabetic macular edema (DME) were included, comprising 20 eyes in the PC-IVF group and 15 eyes in the IVF-PC group. Baseline characteristics are summarized in Table 1. At baseline, the WA was 2.4 ± 1.3 mm^2^ in the PC-IVF group and 2.9 ± 1.2 mm^2^ in the IVF-PC group. The number of microaneurysms (MAs) within the WA was 16.3 ± 7.1 and 19.7 ± 8.0, respectively. There were no significant differences between the groups in baseline WA, MA number, or macular volume.

### 3.2. Visual and Anatomical Outcomes

**Figure 1:**
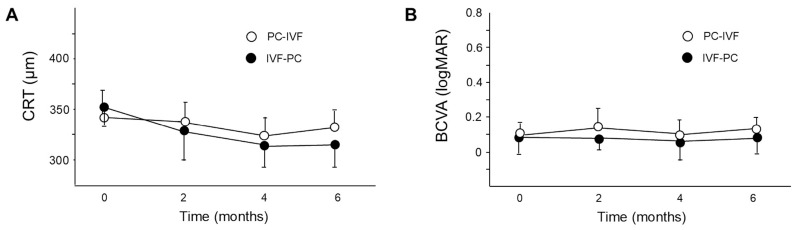
Changes in central retinal thickness (CRT) (**A**) and best-corrected visual acuity (BCVA) in logMAR (**B**) after treatment. Data are presented as mean ± standard deviation (SD).

**Figure 2:**
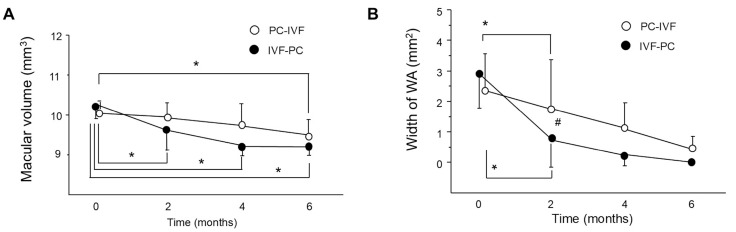
Early anatomical response differs according to treatment sequence. (**A**) Changes in macular volume. (**B**) Width of the white area (WA) on optical coherence tomography (OCT) after treatment. Data are presented as mean ± standard deviation (SD). * *p* < 0.05 (versus each time point), # *p* < 0.05 (IVF-PC group versus PC-IVF group).

No significant differences in central retinal thickness or BCVA were observed between the two groups at any time point (Figure 1A,B). Macular volume significantly decreased after 2, 4, and 6 months in the IVF-PC group (all *p* < 0.01), whereas a significant reduction was observed only after 6 months in the PC-IVF group (*p* < 0.01) (Figure 2A). The WA width significantly decreased from baseline after 2 months in both groups (PC-IVF: *p* = 0.0015; IVF-PC: *p* = 0.0004) and remained reduced thereafter (both *p* < 0.0001). After 2 months, the WA was significantly smaller in the IVF-PC group than in the PC-IVF group (*p* = 0.0291) (Figure 2B).

### 3.3. Treatment Outcomes

**Figure 3:**
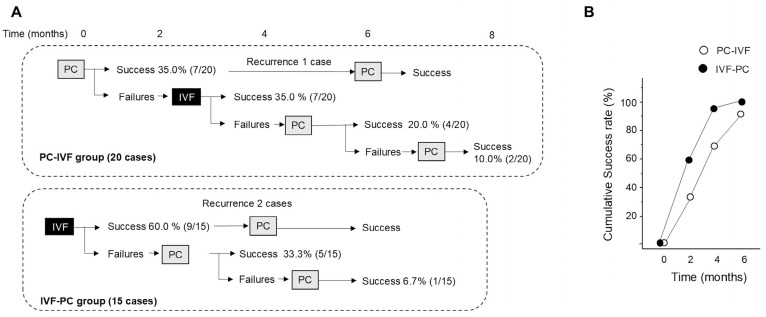
(**A**) Treatment algorithm and outcomes according to treatment sequence for patients with focal diabetic macular edema. Each branch indicates a decision point leading to either intravitreal faricimab (IVF) injection, direct photocoagulation (PC), or observation, depending on the clinical outcome (Success or Failure). “Success” was defined as complete resolution of focal edema, indicated by disappearance of the white area (WA) on OCT color maps, and “Failure” as persistence of edema requiring additional treatment. (**B**) Temporal profile of the cumulative success rate in the IVF-PC and PC-IVF groups.

**Figure 4:**
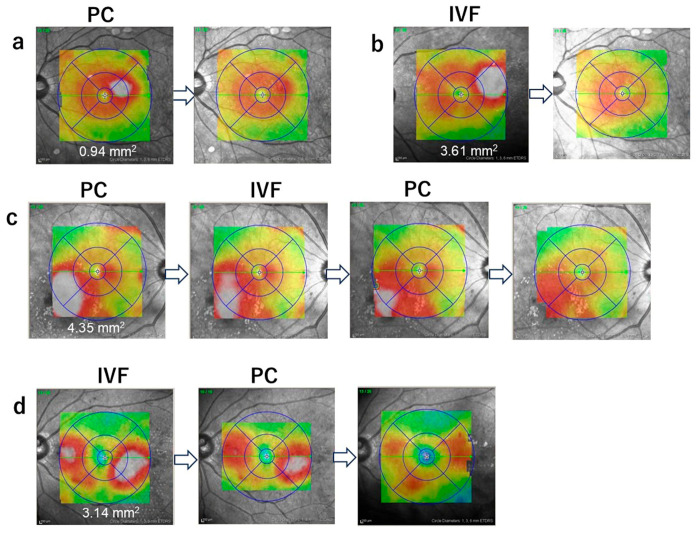
Representative cases showing clinical courses achieving success, indicated by the disappearance of the white area (WA). (**a**) Direct PC alone; (**b**) IVF alone; (**c**) PC followed by IVF, then additional PC; (**d**) IVF followed by PC.

The treatment algorithm and outcomes according to treatment sequence for patients with focal diabetic macular edema are shown in Figure 3A. The cumulative success rates (95% confidence intervals) after 2, 4, and 6 months were 35.0% (15.4–59.2), 70.0% (45.7–88.1), and 90.0% (68.3–98.8) in the PC-IVF group and 60.0% (32.2–83.7), 93.3% (68.0–99.8), and 100% (78.2–100) in the IVF-PC group, respectively (Figure 3B). In the PC-IVF group, 35.0% (7/20) of eyes achieved success after 2 months after the initial PC. The remaining eyes received IVF, after which additional PC was performed in cases of persistent edema. In the IVF-PC group, 60.0% (9/15) of eyes achieved success after initial IVF. Among the remaining eyes, PC was performed, resulting in resolution in most of these cases. One case required an additional PC session to achieve success. After treatment switching, recurrence of DME occurred in 1 eye in the PC-IVF group and 2 eyes in the IVF-PC group, and success was achieved after additional PC. The mean number of PC sessions was lower in the IVF-PC group (0.6) than in the PC-IVF group (1.8). Representative cases that achieve the success after treatment are shown in Figure 4.

### 3.4. Microaneurysm Reduction and Laser Requirement

**Figure 5:**
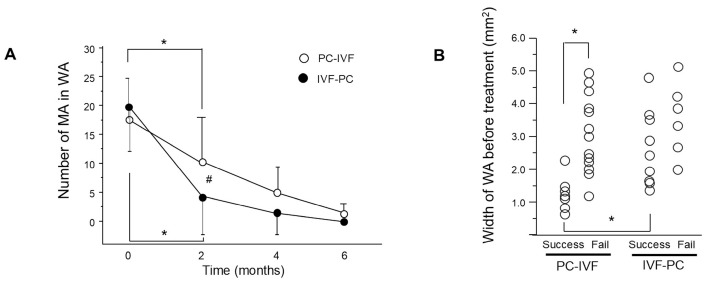
Reduction in laser burden following initial faricimab treatment. (**A**) Histogram showing the number of laser shots during initial photocoagulation (PC) in the PC-intravitreal faricimab (IVF) and IVF-PC groups. Data are presented as mean ± standard deviation (SD). * *p* < 0.05. (**B**) Comparison of baseline white area (WA) width between success and failure cases after initial treatment in the PC-IVF and IVF-PC groups. * *p* < 0.05 (versus each time point). # *p* < 0.05 (IVF-PC group versus PC-IVF group).

The number of MAs within the WA significantly decreased after 2 months in both groups (both *p* = 0.0006) and remained reduced thereafter (Figure 5A). After 2 months, the MA count was significantly lower in the IVF-PC group than in the PC-IVF group (*p* = 0.044). The number of laser shots required for initial PC was significantly higher in the PC-IVF group (18.4 ± 8.1) than in the IVF-PC group (5.6 ± 6.8; *p* < 0.0001).

### 3.5. Predictors of Treatment Response

In the PC-IVF group, the baseline WA was significantly smaller in eyes that achieved success after initial PC than in those that did not (*p* = 0.0005). In contrast, no significant association between baseline WA and treatment success was observed in the IVF-PC group. Among successfully treated eyes, the baseline WA was significantly smaller in the PC-IVF group than in the IVF-PC group (*p* = 0.0022) (Figure 5B).

### 3.6. Safety

No treatment-related adverse events, including vitreous hemorrhage or endophthalmitis, were observed during the study period.

**Figure 6:**
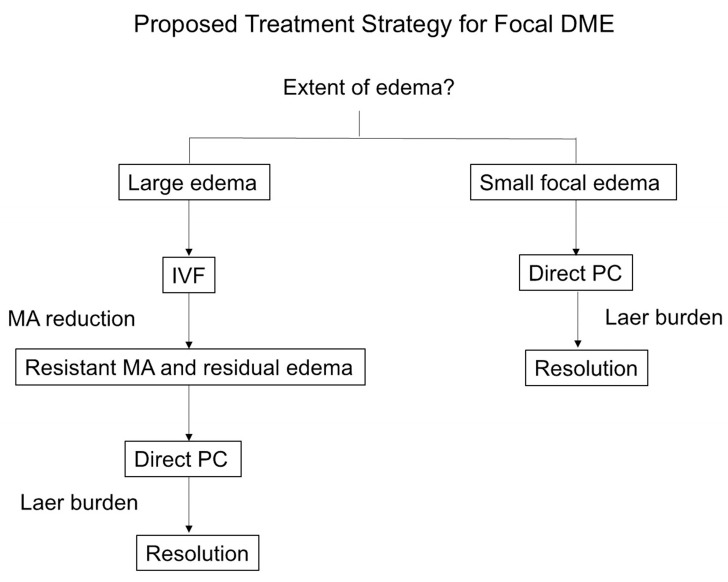
Proposed treatment strategy for focal diabetic macular edema (DME). In eyes with large areas of focal edema, initial intravitreal faricimab (IVF) reduces microaneurysm (MA) burden (MA reduction), thereby facilitating subsequent direct photocoagulation (PC) with fewer laser shots (reduced laser burden). In contrast, in eyes with small focal edema, direct PC alone may be sufficient.

Based on these findings, we developed a conceptual treatment scheme illustrating the differential impact of treatment sequence according to edema size (Figure 6). In eyes with larger edema, initial IVF was associated with a reduction in microaneurysm burden, facilitating subsequent targeted photocoagulation with fewer laser shots, whereas in eyes with smaller edema, direct photocoagulation alone was often sufficient to achieve resolution.

## 4. Discussion

In this study, we demonstrated that the treatment sequence of IVF and direct photocoagulation (PC) significantly influences anatomical outcomes in focal diabetic macular edema (DME). The cumulative success rate was consistently higher in the IVF-PC group than in the PC-IVF group throughout the observation period. In addition, macular volume decreased earlier, and both the WA and MA counts were significantly reduced at an earlier stage in the IVF-PC group. These findings suggest that initiating treatment with IVF facilitates more rapid resolution of focal edema.

The superiority of the IVF-first strategy may be explained by its effect on MA dynamics. In the IVF-PC group, the number of MAs within the edematous region markedly decreased after initial treatment, which in turn reduced the number of laser shots required for subsequent PC. Because MAs represent the primary source of leakage in focal DME, their reduction likely enhances the efficiency of targeted laser therapy. In contrast, when PC is performed first in eyes with extensive edema, numerous MAs must be treated individually, which may limit the overall effectiveness of the procedure.

Faricimab is a bispecific antibody that inhibits both VEGF-A and Ang-2, thereby targeting complementary pathways involved in vascular permeability and instability. Ang-2 is known to destabilize retinal vasculature and promote pericyte loss, contributing to MA formation and persistence [20,21,22]. By simultaneously inhibiting VEGF-A and Ang-2, faricimab may promote vascular stabilization, reduce pericyte loss, and facilitate regression of MAs. Consistent with this mechanism, we previously demonstrated a substantial reduction in MA number following IVF [19]. The present findings further support the concept that IVF not only reduces vascular permeability but also modifies the structural basis of focal edema, thereby improving the efficacy of subsequent PC.

Nevertheless, persistent edema may remain in some cases even after repeated IVF, and we have previously reported that residual edema is frequently associated with larger MAs [22]. These findings suggest that large MAs may contribute to resistance to faricimab. In contrast, larger MAs may be advantageous targets for direct PC, as they can be more readily identified and accurately coagulated. Therefore, a treatment strategy in which IVF reduces the overall MA burden, allowing the identification of faricimab-resistant MAs, followed by selective elimination of these lesions using direct PC, may represent a rational approach to overcoming the limitations of faricimab.

Despite these benefits, direct PC remains an effective treatment option in selected cases. In the PC-IVF group, baseline WA was significantly smaller in eyes that achieved success after initial PC, indicating that PC alone may be sufficient for limited focal edema. However, this observation is based on a post hoc analysis and should be interpreted with caution. Given the high cost and treatment burden associated with anti-VEGF therapy, an individualized approach based on edema size may be clinically relevant. For small focal lesions, direct PC may provide a cost-effective and less invasive alternative without the need for intravitreal injections.

Another clinically relevant finding is the reduction in treatment burden with the IVF-first approach. The numbers of laser shots initially performed and sessions of additional PC were significantly lower in the IVF-PC group, suggesting reduced retinal invasiveness. A lower number of laser applications may also minimize cumulative retinal damage and scarring, which is particularly relevant in preserving retinal function. Moreover, a substantial proportion of eyes achieved resolution with IVF alone, obviating the need for additional laser treatment. These advantages may contribute to improved patient comfort and adherence in real-world clinical settings.

Another important consideration is that only a single IVF was administered prior to treatment switching, whereas current practice often involves a loading phase or treat-and-extend regimen. A more intensive anti-VEGF approach may have further enhanced edema resolution and reduced the need for subsequent PC. However, in the present study, a substantial reduction in MA burden was observed after a single injection, suggesting that even one dose may be sufficient for lesion modulation in many cases. This finding is consistent with our previous report demonstrating early MA regression following IVF. Nevertheless, further studies directly comparing single- and multi-injection regimens are warranted.

The lack of significant improvement in BCVA observed in this study may be attributable to the non-center-involved nature of the edema, as the foveal center was relatively preserved at baseline. In addition, the relatively short follow-up period may have limited the ability to detect functional changes despite anatomical improvement.

This study has several limitations. First, its retrospective design and relatively small sample size may introduce selection bias. Because treatment allocation was based on different time periods, potential temporal bias cannot be completely excluded. Second, the anti-VEGF regimen was limited, and a more intensive protocol (e.g., loading or treat-and-extend) might have further improved edema resolution and reduced the need for PC. Third, the follow-up period was limited to 6 months, and longer-term outcomes, including recurrence rates, remain unclear. Future prospective, randomized controlled trials with larger sample sizes and a longer follow-up are warranted to validate our findings.

In conclusion, initiating treatment with IVF prior to direct PC appears to be an effective strategy for managing focal DME, particularly in eyes with larger areas of edema. This approach enables faster anatomical improvement, reduces the need for laser treatment, and may decrease overall treatment burden. Conversely, direct PC alone may be sufficient for small focal lesions, highlighting the importance of individualized treatment selection based on disease extent. These findings support a stepwise treatment algorithm and provide a clinically applicable framework for optimizing therapeutic decision-making in focal DME. This strategy may contribute to a paradigm shift from uniform treatment toward lesion-oriented, precision-based management of focal DME.

## Figures and Tables

**Table 1:** Baseline characteristics of study patients.

Parameter	PC-IVF Group	IVF-PC Group	*p* Value
	(*n* = 20)	(*n* = 15)	
Age (years)	64.1 ± 6.8	62.1 ± 7.9	0.059 *
Sex (male/female)	13/7	9/6	0.76 ^†^
Duration of DM (years)	5.34 ± 6.09	4.53 ± 3.26	0.74 *
Hemoglobin A1c (%)	7.83 ± 1.25	7.42 ± 0.89	0.44 *
Insulin therapy	7 (35.0%)	6 (40.0%)	0.76 ^†^
Left eye: right eye	11:9	8:7	0.92 ^†^
Creatinine (mg/dL)	0.91 ± 0.28	0.88 ± 0.31	0.29 *
Mild ~ moderate NPDR	18 (90.0%)	12 (80.0%)	0.62 ^†^
Severe NPDR	2 (10.0%)	3 (20.0%)	0.62 ^†^

* Unpaired *t*-test; ^†^ Fisher’s exact test; IVF, intravitreal injection of faricimab; PC, photocoagulation; DM, diabetes mellitus; NPDR, non-proliferative diabetic retinopathy.

## Data Availability

The data presented in this study are available from the corresponding author upon reasonable request.

## References

[1] Im J.H.B., Jin Y.P., Chow R., Yan P. (2022). Prevalence of Diabetic Macular Edema Based on Optical Coherence Tomography in People with Diabetes: A Systematic Review and Meta-Analysis. Surv. Ophthalmol..

[2] Browning D.J., Altaweel M.M., Bressler N.M., Bressler S.B., Scott I.U., Diabetic Retinopathy Clinical Research Network (2008). Diabetic Macular Edema: What Is Focal and What Is Diffuse?. Am. J. Ophthalmol..

[3] Murakami T., Nishijima K., Sakamoto A., Ota M., Horii T., Yoshimura N. (2011). Foveal Cystoid Spaces Are Associated with Enlarged Foveal Avascular Zone and Microaneurysms in Diabetic Macular Edema. Ophthalmology.

[4] Lee S.N., Chhablani J., Chan C.K., Wang H., Barteselli G., El-Emam S., Gomez M.L., Kozak I., Cheng L., Freeman W.R. (2013). Characterization of Microaneurysm Closure after Focal Laser Photocoagulation in Diabetic Macular Edema. Am. J. Ophthalmol..

[5] Early Treatment Diabetic Retinopathy Study Research Group (1995). Focal Photocoagulation Treatment of Diabetic Macular Edema: Relationship of Treatment Effect to Fluorescein Angiographic and Other Retinal Characteristics at Baseline: ETDRS Report No. 19. Arch. Ophthalmol..

[6] Ogura Y., Shiraga F., Terasaki H., Ohji M., Ishida S., Sakamoto T., Hirakata A., Ishibashi T. (2017). Clinical Practice Pattern in Management of Diabetic Macular Edema in Japan: Survey Results of Japanese Retinal Specialists. Jpn. J. Ophthalmol..

[7] Kato F., Nozaki M., Kato A., Hasegawa N., Morita H., Yoshida M., Ogura Y. (2018). Evaluation of Navigated Laser Photocoagulation (Navilas 577+) for the Treatment of Refractory Diabetic Macular Edema. J. Ophthalmol..

[8] Nozaki M., Kato A., Yasukawa T., Suzuki K., Yoshida M., Ogura Y. (2019). Indocyanine Green Angiography-Guided Focal Navigated Laser Photocoagulation for Diabetic Macular Edema. Jpn. J. Ophthalmol..

[9] Kozak I., Oster S.F., Cortes M.A., Dowell D., Hartmann K., Kim J.S., Freeman W.R. (2011). Clinical Evaluation and Treatment Accuracy in Diabetic Macular Edema Using Navigated Laser Photocoagulator NAVILAS. Ophthalmology.

[10] Ikegami Y., Shiraya T., Araki F., Ueta T., Toyama T., Yanagita T., Numaga J., Shoji N., Kato S. (2023). Navigated Direct Photocoagulation with a 30-Ms Short-Pulse Laser for Treating Microaneurysms in Diabetic Macular Edema Exhibits a High Closure Rate. Sci. Rep..

[11] Terasaki H., Ogura Y., Kitano S., Sakamoto T., Murata T., Hirakata A., Ishibashi T. (2018). Management of Diabetic Macular Edema in Japan: A Review and Expert Opinion. Jpn. J. Ophthalmol..

[12] Mitchell P., Bandello F., Schmidt-Erfurth U., Lang G.E., Massin P., Schlingemann R.O., Sutter F., Simader C., Burian G., Gerstner O. (2011). The RESTORE Study: Ranibizumab Monotherapy or Combined with Laser versus Laser Monotherapy for Diabetic Macular Edema. Ophthalmology.

[13] Glassman A.R., Wells J.A., Josic K., Maguire M.G., Antoszyk A.N., Baker C., Beaulieu W.T., Elman M.J., Jampol L.M., Sun J.K. (2020). Five-Year Outcomes after Initial Aflibercept, Bevacizumab, or Ranibizumab Treatment for Diabetic Macular Edema (Protocol T Extension Study). Ophthalmology.

[14] Bressler N.M., Beaulieu W.T., Glassman A.R., Blinder K.J., Bressler S.B., Jampol L.M., Melia M., Wells J.A. (2018). Persistent Macular Thickening Following Intravitreous Aflibercept, Bevacizumab, or Ranibizumab for Central-Involved Diabetic Macular Edema with Vision Impairment: A Secondary Analysis of a Randomized Clinical Trial. JAMA Ophthalmol..

[15] Yamada Y., Takamura Y., Matsumura T., Gozawa M., Morioka M., Inatani M. (2022). Regional Variety of Reduction in Retinal Thickness of Diabetic Macular Edema after Anti-VEGF Treatment. Medicina.

[16] Yamada Y., Takamura Y., Morioka M., Gozawa M., Matsumura T., Inatani M. (2021). Microaneurysm Density in Residual Oedema after Anti-Vascular Endothelial Growth Factor Therapy for Diabetic Macular Oedema. Acta Ophthalmol..

[17] Chaudhary V., Mar F., Amador M.J., Chang A., Gibson K., Joussen A.M., Kim J.E., Lee J., Margaron P., Saffar I. (2025). Emerging Clinical Evidence of a Dual Role for Ang-2 and VEGF-A Blockade with Faricimab in Retinal Diseases. Graefe’s Arch. Clin. Exp. Ophthalmol..

[18] Wykoff C.C., Abreu F., Adamis A.P., Basu K., Eichenbaum D.A., Haskova Z., Lin H., Loewenstein A., Mohan S., Pearce I.A. (2022). Efficacy, Durability, and Safety of Intravitreal Faricimab with Extended Dosing up to Every 16 Weeks in Patients with Diabetic Macular Oedema (YOSEMITE and RHINE): Two Randomised, Double-Masked, Phase 3 Trials. Lancet.

[19] Takamura Y., Yamada Y., Morioka M., Gozawa M., Matsumura T., Inatani M. (2023). Turnover of Microaneurysms After Intravitreal Injections of Faricimab for Diabetic Macular Edema. Investig. Ophthalmol. Vis. Sci..

[20] Benest A.V., Kruse K., Savant S., Thomas M., Laib A.M., Loos E.K., Fiedler U., Augustin H.G. (2013). Angiopoietin-2 Is Critical for Cytokine-Induced Vascular Leakage. PLoS ONE.

[21] Cai J., Kehoe O., Smith G.M., Hykin P., Boulton M.E. (2008). The Angiopoietin/Tie-2 System Regulates Pericyte Survival and Recruitment in Diabetic Retinopathy. Investig. Ophthalmol. Vis. Sci..

[22] Park S.W., Yun J.H., Kim J.H., Kim K.W., Cho C.H., Kim J.H. (2014). Angiopoietin 2 Induces Pericyte Apoptosis via A3β1 Integrin Signaling in Diabetic Retinopathy. Diabetes.

